# Adolescent Basic Facial Emotion Recognition Is Not Influenced by Puberty or Own-Age Bias

**DOI:** 10.3389/fpsyg.2018.00956

**Published:** 2018-06-21

**Authors:** Nora C. Vetter, Mandy Drauschke, Juliane Thieme, Mareike Altgassen

**Affiliations:** ^1^Department of Child and Adolescent Psychiatry, Faculty of Medicine, Technische Universität Dresden, Dresden, Germany; ^2^Department of Psychiatry and Neuroimaging Center, Technische Universität Dresden, Dresden, Germany; ^3^Department of Psychology, Technische Universität Dresden, Dresden, Germany; ^4^Department of Psychology, Bergische Universität Wuppertal, Wuppertal, Germany; ^5^Donders Institute for Brain, Cognition and Behaviour, Radboud University Nijmegen, Nijmegen, Netherlands

**Keywords:** adolescence, puberty, emotion, pubertal dip, own-age bias, development

## Abstract

Basic facial emotion recognition is suggested to be negatively affected by puberty onset reflected in a “pubertal dip” in performance compared to pre- or post-puberty. However, findings remain inconclusive. Further, research points to an own-age bias, i.e., a superior emotion recognition for peer faces. We explored adolescents’ ability to recognize specific emotions. Ninety-five children and adolescents, aged 8–17 years, judged whether the emotions displayed by adolescent or adult faces were angry, sad, neutral, or happy. We assessed participants *a priori* by pubertal status while controlling for age. Results indicated no “pubertal dip”, but decreasing reaction times across adolescence. No own-age bias was found. Taken together, basic facial emotion recognition does not seem to be disrupted during puberty as compared to pre- and post-puberty.

## Introduction

The transitional age phase of adolescence can roughly be defined as ranging from early (11–13 years) over middle (14–17 years) until late adolescence/ emerging adulthood (18–22 years; Arnett, 2000; Steinberg, 2008). Adolescence is defined socioculturally, while puberty is defined as the process of physical changes that lead to sexual maturation. Key-developmental tasks in adolescence are forming more complex peer relationships, first romantic relationships and the detachment from parents (Lerner and Steinberg, 2004). Well-functioning basic facial emotion recognition is therefore crucial for the transitional phase of adolescence. Adolescents need to be able to quickly and accurately identify the facial emotions of their parents and peers in order to act appropriately. For instance, adolescents first need to identify if their mother is angry or their best friend is sad to respond accordingly and try to calm down the mother or to cheer up the best friend.

Empirical evidence indicates that, even though basic facial emotion recognition starts to develop in infancy (Nelson, 1987), children continue to become faster and more accurate in decoding others’ basic facial emotions until adolescence (Kolb et al., 1992; De Sonneville et al., 2002; Herba and Phillips, 2004; Gao and Maurer, 2010; Chronaki et al., 2015), and this development seems to continue across adolescence until adulthood. In fact, there is evidence for an ongoing development of basic facial emotion recognition until young adulthood from two lines of research: First, *face* recognition, which may underlie the development of basic facial *emotion* recognition (De Sonneville et al., 2002), continues to develop across adolescence into early adulthood (Chung and Thomson, 1995; Germine et al., 2011; Meinhardt-Injac et al., 2014). Second, the neural networks that process (emotional) faces show continued structural and functional specialization across adolescence (Monk et al., 2003; Monk, 2008; Guyer et al., 2008; Golarai et al., 2010). However, surprisingly, as already noted by Scherf and Scott (2012), there is no systematic theoretical framework on possible underlying cognitive or neural mechanisms that drive the development of basic facial emotion recognition across childhood and adolescence.

While overall empirical evidence from related studies (face recognition, neural development) may point to an ongoing development of basic facial emotion recognition across adolescence to young adulthood, the shape of this trajectory is unclear. Two studies have suggested a steady linear development of basic facial emotion recognition (Herba et al., 2006; Montirosso et al., 2010), while two others have reported a non-linear trajectory with a decrease of performance at the onset of puberty followed by a regain to the previous level in post-puberty (McGivern et al., 2002; Tonks et al., 2007). The underlying mechanisms of this so-called “pubertal dip” are unclear (for a review see also Blakemore, 2008). It has been hypothesized that this dip is due to less efficient neural regions for facial processing during adolescence (first suggested by Carey et al., 1980, here related to identity recognition). Scherf and Scott (2012), on the other hand, postulate that the developmental task of re-orientation from parents toward peers (Forbes and Dahl, 2010; Scherf et al., 2012) may cause a temporal disruption of existing facial (emotion) processing abilities at puberty onset (see also Picci and Scherf, 2016).

Given these inconsistent findings, the present study set out to further determine whether this “pubertal dip” actually exists. Only two studies so far have indirectly found this dip in basic facial emotion recognition (McGivern et al., 2002; Tonks et al., 2007), and both suffered from some methodological limitations. Neither study measured pubertal status, but used age as a proxy, which is only a very limited approach given that the beginning of puberty largely differs between individuals (Petersen et al., 1988). Both studies assessed 10–12 year-olds, which were expected to be pubertal, and results showed lower basic facial emotion recognition than adjacent age groups across emotions (McGivern et al., 2002; Tonks et al., 2007). McGivern et al. (2002) used a task in which participants had to match a given emotional word to a facial expression (happy, sad, angry, and neutral); here, reaction times were measured. Tonks et al. (2007) assessed error rates for five facial expression tests of the Florida affect battery (e.g., naming, selection, matching for happy, sad, angry, frightened, and neutral emotions).

More indirect evidence on the related ability of *face* recognition has been provided by two studies. One early study reported lower face recognition for pubertal girls (here, pubertal status was assessed by physical exam; Diamond et al., 1983). Another recent study found that pre-pubertal children show better recognition of adult female faces relative to child and adolescent faces, whereas during puberty this bias shifted and peer faces (versus adult and child faces) were better recognized (Picci and Scherf, 2016). In contrast, other studies on face recognition point toward a linear development without any disruption in adolescence (Germine et al., 2011; Meinhardt-Injac et al., 2014). Meinhardt-Injac et al. (2014) investigated same-different matching accuracy by asking children (8–10 years), adolescents (11–16 years), and adults to compare the identity of two faces. They found ongoing improvement until young adulthood. Germine et al. (2011) assessed face recognition with an online face memory task (Cambridge Face Memory Test) in participants from 10 to 70 years and found that performance peaks at about age 30.

Two other studies that directly tested basic facial emotion recognition reported a continuous increase of basic facial emotion recognition across adolescence (Herba et al., 2006; Montirosso et al., 2010). Both studies assessed accuracy of recognizing expressions of anger, sadness, happiness, fear, and disgust across adolescence. Herba et al. (2006) asked participants to match the emotion of a target face to one of two faces, while Montirosso et al. (2010) assessed the ability to name an emotion after a short sequence of animated faces with varying emotional intensities. In contrast to the studies that found a pubertal dip, these studies assessed a wider age range (Herba et al., 2006: 4–15 years; Montirosso et al., 2010: 10–18 years). However, similarly to the McGivern et al. (2002) and Tonks et al. (2007) studies, neither study assessed pubertal status of participants which limits their conclusions.

Taken together, the ongoing debate on a possible pubertal dip remains unresolved. Therefore, the present study set out to systematically investigate the developmental trajectory of basic facial emotion recognition across pubertal stages. To overcome previous methodological limitations, we assessed pubertal status *a priori* to be able to compare similar sample sizes of pubertal groups.

As indicated above, a recent review has suggested that the pubertal dip of facial (emotion) recognition may follow a new emphasis on peers’ faces. This may in turn lead to a reorganization of the face processing system indicated by a superior accuracy for own-age versus other age faces (own-age bias; Scherf and Scott, 2012). So far, only one study has directly tested this assumption in adolescents and found supporting evidence (Picci and Scherf, 2016); with children being better at recognizing adult faces, whereas during and after puberty own-age (peer) faces were better recognized (for comparable evidence at the other end of the age span see Rhodes and Anastasi, 2012). In contrast, Griffiths et al. (2015) did not find an own-age bias in basic facial emotion recognition for happy, sad, angry, surprised, fearful, and disgusted faces in a multiple choice task in children aged 5–13 and interpreted this result with the extensive experience of children with adults (teachers and parents). Interpretation of this study with regard to the framework of Scherf and Scott (2012) and Picci and Scherf (2016) is limited since puberty was not assessed.

However, the vast majority of studies on basic facial emotion recognition in adolescents have employed adult faces and neglected age-matched stimuli (De Sonneville et al., 2002; McGivern et al., 2002; Durand et al., 2007; Lawrence et al., 2015; Rodger et al., 2015).

Importantly, the phenomenon of an own-age bias may not only be present in children and adolescents, but also in adults. For example, own-age biases were observed in young, middle-aged, and older adult women with regards to basic facial emotion recognition of anger, sadness, and fear (Malatesta et al., 1987). Similarly, for younger and older adults (Riediger et al., 2011) an own-age bias was shown for the emotions of happiness and anger. Possibly, the own-age bias results from more frequent contact with members of the own-age group that leads to enhanced perceptual expertise or to greater motivation to more closely look at own-age faces (Rhodes and Anastasi, 2012). However, not all studies point toward the presence of an own-age bias. For example, Ebner and Johnson (2009) and Ebner et al. (2011) reported no own-age bias in adults when they were requested to identify the emotions happiness, anger, fear, sadness, disgust, and neutrality using a multiple choice paradigm.

Given that the own-age bias might be closely linked to a reorientation toward peers (Scherf and Scott, 2012), and a possible pubertal dip the current study set out to explore whether there is an own-age bias for adolescents in recognizing basic facial emotions of peers and adults.

Our third aim was to explore the possible differential recognition of specific positive and negative basic facial emotions across adolescence. Positive emotions are recognized first in ontogeny, and more accurately across development than negative emotions (Felleman et al., 1983; Kolb et al., 1992; Boyatzis and Chazan, 1993; Durand et al., 2007). Specifically, happiness seems to be recognized from a very young age on (Felleman et al., 1983; Durand et al., 2007). In contrast, findings on negative emotions are rather inconsistent. While some studies have indicated correct identification of sadness early in development (Felleman et al., 1983; Durand et al., 2007), others have reported low accuracy until about 14 years (Kolb et al., 1992; Montirosso et al., 2010). Whether sadness (Boyatzis and Chazan, 1993) or anger (Montirosso et al., 2010; Chronaki et al., 2015) are easier to recognize in childhood and adolescence also remains inconclusive. Anger seems to be a particularly difficult emotion to recognize in childhood (Boyatzis and Chazan, 1993); and reduced performance as compared to adults has even been found in adolescents (Thomas et al., 2007). The prolonged adolescent development of negative emotions might be related to the ongoing development of the neural network that supports emotion recognition (Grosbras and Paus, 2006) which also involves the prefrontal cortex (Casey et al., 2008). For example, anger is a self-conscious and social emotion and its expression underlies cultural norms (Berkowitz, 1999) which may require cognitive control resources that rely on the prefrontal cortex (Thomas et al., 2007). Further, children and adolescents seem to have difficulties to detect neutrality (Rodger et al., 2015) and often mistake it for happiness or sadness (Durand et al., 2007). Summarizing these results, the development of basic facial emotion recognition seems to depend on emotions’ valence, with positive emotions being recognized generally better and earlier in development than negative or neutral emotions. Therefore, some basic (negative) emotions might be more difficult to detect and differentiate in adolescence than basic positive emotions. The emotional categories might be more poorly defined and adolescents’ difficulties in recognizing negative emotions might stem from less knowledge about their effects on facial expressions (Durand et al., 2007). This might play a role in adolescents’ relationships and interactions with peers and adults. Adolescents might have difficulties to interact socially adequate when specific (negative) emotions are expressed.

The first aim of the current study was to systematically investigate the development of basic facial emotion recognition across puberty. Given the reorientation framework by Scherf and Scott (2012), the study of Picci and Scherf (2016), and previous reports of a pubertal dip in adolescence (Diamond et al., 1983; McGivern et al., 2002) we hypothesized that pubertal adolescents would show a lower basic facial emotion recognition for adult faces compared to pre-pubertal and post-pubertal adolescents in reaction times and accuracy. Our second aim was to explore a potential own-age bias for basic facial emotion recognition in adolescence. Based on Scherf and Scott’s (2012) theory that there is a new emphasis on peers’ faces at the onset of puberty as well as studies reporting an own-age bias in adults (Malatesta et al., 1987; Riediger et al., 2011), we hypothesized that pre-pubertal adolescents would show better basic facial emotion recognition for adult faces, while adolescents after the onset of puberty (pubertal and post-pubertal) would show a higher recognition of adolescent faces. The third aim of this study was to explore the recognition of different types of emotions in adolescence. We expected that positive (happy) emotions would be recognized faster and more accurately than negative (angry and sad) and neutral emotions across adolescence (Kolb et al., 1992; Montirosso et al., 2010). We chose these specific emotions to closely match emotions used by McGivern et al. (2002) who found a pubertal dip in basic facial emotion recognition while using age as a proxy for puberty.

## Materials and Methods

### Participants

#### Recruiting and Testing Procedure

Adolescents were recruited via flyers in high schools, primary schools, or youth centers. Participants were in 3rd to 10th grade and mostly attended a higher grammar school (German “Gymnasium”). They were tested indivicually at school and received a small present for participation and took part in a lottery for cinema vouchers. The first 20 participants were assessed without *a priori* testing for pubertal status because at this stage it was not critical which pubertal status groups were recruited first. These first twenty participants started the testing session with the verbal ability task followed by the emotion recognition task and a non-verbal ability test. At the end of the session, participants filled in the sociodemographic questionnaires and the Pubertal Developmental Scale (PDS). After assessing these first 20 participants, to ensure similar sizes of each pubertal status group (pre-pubertal, pubertal, and post-pubertal), during the recruitment process and before participation in the study we first assessed pubertal status via an online questionnaire, then allocated potential participants to one of the three groups and assessed them. When groups were filled (e.g., pre-pubertal boys), we did not invite male participants that had the same pubertal status according to the online questionnaire. Although we tried to ensure similar sample sizes regarding pubertal status and gender, this was not perfectly possible. For the following 75 participants during the testing session, the study authors first assessed participants with regards to their verbal abilities followed by the emotion recognition task. Then participants performed the non-verbal ability test. At the end of the visit, participants filled in the sociodemographic questionnaires. Instructions for the emotion recognition task were computerized and experimenters provided standardized instructions for this task.

#### Sample

The total sample comprised 95 Caucasian participants (see **Table 1**): Thirty-three pre-pubertal adolescents (14 female) aged 8–13 years (*M* = 10.82, *SD* = 1.21), 31 pubertal adolescents (16 female) aged 8–16 years (*M* = 11.77, *SD* = 1.63), and 31 post-pubertal adolescents (22 female) aged 10–17 years (*M* = 13.94, *SD* = 1.79). By design, groups differed with regard to age, *F*(2,92) = 31.8, *p* < 0.001, with age increasing from the pre-pubertal to the post-pubertal group. There were no significant differences with respect to gender distribution across groups, χ^2^ (2, *N* = 95) = 5.38, *p* = 0.068. All participants were native German speakers. Exclusion criteria were psychiatric disorders such as attention-deficit hyperactivity disorder, depression, mania, or schizophrenia (as assessed by parental report). No recruited participant had to be excluded due to exclusion criteria or technical difficulties. All participants and their parents or guardians gave written informed consent prior to testing. The study was conducted in compliance with the Declaration of Helsinki and was approved by the local ethics committee.

**Table 1:** Means and (Standard Deviations) of sample characteristics sorted by gender and pubertal status.

	Girls			Boys			Comparison of pubertal groups including both genders
Pubertal status	Pre-pubertal	Pubertal	Post-pubertal	Pre-pubertal	Pubertal	Post-pubertal	
*n*	14	16	22	19	15	9	*p* < 0.001, pre-pubertal < pubertal < post-pubertal
Age	10.29 (0.99)	10.88 (1.50)	13.50 (1.77)	11.21 (1.23)	12.73 (1.16)	15.00 (1.41)	*p* < 0.001, pre-pubertal < pubertal < post-pubertal
Socioeconomic status^a^	12.00 (4.03)	14.81 (4.24)	13.36 (4.01)	14.29 (4.67)	14.79 (3.27)	15.75 (3.85)	n.s.
Parental cultural capital^b^	2.07 (0.62)	2.44 (0.63)	2.23 (0.69)	2.32 (0.75)	2.43 (0.51)	2.75 (0.46)	n.s.
Non-verbal abilities	11.43 (2.14)	11.62 (2.9)	10.32 (2.21)	10.42 (2.91)	11.33 (2.5)	11.00 (2.9)	n.s.
Verbal abilities	11.79 (1.67)	13.25 (2.18)	11.09 (2.20)	13.37 (2.36)	11.67 (1.76)	10.78 (1.64)	*p* < 0.01, pubertal > post-pubertal, pre-pubertal > post-pubertal

*^*a*^as suggested by Winkler and Stolzenberg (2009) ranging from 3 to 21 with higher values indicating higher socioeconomic status; ^*b*^1* = *lower class, 2* = *middle class, 3*= *upper class (De Graaf et al., 2000)*.

Following Winkler and Stolzenberg’s (2009) suggestion, socioeconomic status was calculated based on parents’ school education, professional education, recent professional status, and family income. Scores for mothers and fathers were averaged into a family-based measure of socioeconomic background and ranged between 3 and 21, with higher values indicating higher socioeconomic status. Socioeconomic status did not differ between groups, *F*(2,92) = 1.02, *p* = 0.366. Additionally, a proxy for parental cultural capital (De Graaf et al., 2000), based on numbers of books in their parents’ house, did not differ between groups, χ^2^ (2, *N* = 95) = 3.99, *p* = 0.136.

Verbal abilities (Vocabulary subtest) and non-verbal abilities (Numbers subtest) were measured with the Wechsler Intelligence Scale for Children (WISC-IV, German adaptation, Petermann and Petermann, 2007). Groups differed in their age-corrected verbal abilities: *F*(2,92) = 5.96, *p* = 0.004. This was driven by lower verbal abilities in post-pubertal, *M* = 11, *SD* = 2.03, compared to both pubertal, *M* = 12.48, *SD* = 2.11, *t*(118) = 0.66, *p* = 0.007; and pre-pubertal adolescents, *M* = 12.7, *SD* = 2.22, *t*(118) = 0.66, *p* = 0.002. Groups did not differ in their age-corrected non-verbal abilities, *F*(2,92) = 1.14, *p* = 0.324.

### Materials

#### Basic Facial Emotion Recognition

**FIGURE 1:**
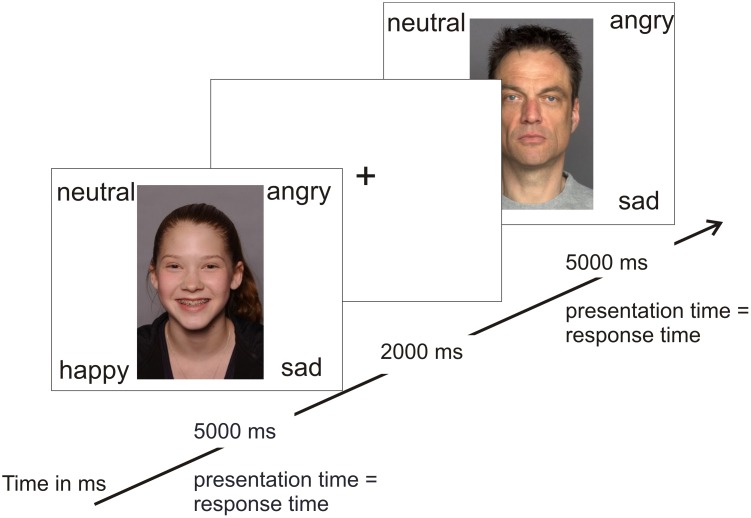
Example trials for the basic facial emotion recognition task.

In order to test the hypothesis of an own-age bias, photographs of angry, neutral, happy, and sad adolescents and adults were taken from two separate databases for emotional faces (see **Figure 1** for stimulus examples). There is currently no database that includes both adult and adolescent stimuli. Photographs of adolescents were taken from the National Institute of Mental Health Child Emotional Faces Picture Set (NIMH-ChEFS) that contains faces of 10–17-year-old adolescents (*M* = 13.6) and has a high validity, *K* = 0.86 (Egger et al., 2011). The NIMH-ChEFS was chosen since it provides facial pictures of the entire age range that was covered by our participants (*M* = 12.7, range 8–17 years). Only pictures with direct eye contact were chosen. This is one of the first studies to evaluate NIMH-ChEFS pictures by adolescents. The NIMH-ChEFS evaluations are comparable with commonly used adult picture sets, including the Ekman Pictures of Facial Affect (Egger et al., 2011). To match our participants’ ethnicity, we first selected Caucasian pictures based on appearance (which build the vast majority of the stimulus set; Egger et al., 2011). The further selection of the photographs resulted from a short pilot study. Five undergraduates were asked to judge which emotion was depicted by the adolescent in the picture. If students’ judgments were incorrect, the picture was excluded. Pictures of middle-aged Caucasian adults (*M* = 49 years, age range 39–55 years) were taken from the FACES Database (Ebner et al., 2010). This age group was chosen to approximate the age of participants’ parents and teachers. Both the younger (*M* = 24 years, age range 19–31) and the older (*M* = 73 years, age range 69–80) age group of the FACES Database seemed inappropriate for this purpose. From each database, 10 happy, 10 neutral, 10 angry, and 10 sad pictures were selected with equal numbers of males and females (5 male and 5 female each). In total, 80 stimuli were shown randomly, i.e., 40 adult and 40 adolescent stimuli. Thus, there were 20 trials per emotion. For details on the stimuli please refer to Supplementary Tables S2, S3.

In random order, stimuli were presented using E-Prime (Psychology Software Tools, Inc.^1^) on a computer screen. Participants sat 50 cm in front of the screen. The four emotion words (happy, angry, sad, and neutral) were presented at each corner of the screen at the same time as the respective emotional stimulus (see **Figure 1**). Each emotion word remained at the same position throughout the task and was related to the same keyboard button to not confuse participants and enable fast responses. Participants were asked to decide as fast and accurately as possible which emotion was being presented by pressing the respective keyboard button. Pictures remained on-screen until a response was made or for a maximum of 5000 ms (all participants answered within this time span). After each trial, a fixation cross was presented for 2000 ms. There was a short break after 40 trials. The total time of the task was 10 min. Participants practiced the same task to get familiarized with the procedure, timing, and the keyboard buttons. The practice task comprised 10 stimuli that were not part of the main task. There was no threshold for continuing to the main task. None of the participants had any difficulties with the practice task. Dependent variables of the main task were (1) the reaction times of correct responses (reaction times of incorrect responses were excluded) and (2) error rates.

#### Pubertal Development

The German version (Watzlawik, 2009) of the self-report questionnaire PDS (Petersen et al., 1988) was used to measure pubertal status. Good reliability and validity data of this scale have been reported (Petersen et al., 1988; Bond et al., 2006). The PDS has been employed in recent studies (Saxbe et al., 2014; Lawrence et al., 2015) and has been shown to be in moderate agreement with physical clinician-ratings and related to basal hormones (Shirtcliff et al., 2009; Saxbe et al., 2014).

The German version of the PDS assesses both adrenarche and gonadarche (see also Shirtcliff et al., 2009) and consists of three questions (for boys: body hair growth, voice change, facial hair growth; for girls: body hair growth, breast development and menarche) assessed on a four points scale from one (maturation not started) to four (maturation completed). Menarche was measured dichotomously as yes (four points) or no (one point). The scores were classified in five status: (1) pre-pubertal, (2) early pubertal, (3) mid-pubertal, (4) late pubertal, and (5) post-pubertal. Following the procedure of Keulers et al. (2010) and Vetter et al. (2013) the five pubertal status were recoded into three status, pre-pubertal (1, 2), pubertal (3), and post-pubertal (4, 5). We collapsed pubertal groups, similarly, as other studies (Keulers et al., 2010; Vetter et al., 2013; Herting et al., 2015; Picci and Scherf, 2016) to achieve similarly distributed groups. We assessed pubertal status in order to be able to compare our results to the target study of Diamond et al. (1983), the only study finding a pubertal dip, which measured puberty and classified adolescents into these three status. The two other studies (McGivern et al., 2002; Tonks et al., 2007) did not measure pubertal status but inferred it only from age as a proxy.

## Results

Descriptively, mean reaction times ranged from 803 to 2347 ms with a mean of 1528 ms (*SD* = 309 ms). Error rates ranged from 13 to 0 errors (percentage correct: 84–100%) with a mean of 5.31 errors (*SD* = 2.74 errors; percentage correct mean: 93.4%, *SD* = 3.4%). For descriptive results of error rates please also refer to Supplementary Tables S4, S5, the original data can also be found online in an excel sheet (Supplementary Data Sheet S1).

### Analytic Approach

Two 3 × 4 × 2 repeated-measures analyses of covariance (ANCOVA) were performed with pubertal status (pre-pubertal, pubertal, and post-pubertal) as a between-subjects variable, and emotion (happy, neutral, angry, and sad), and stimulus age (adolescent stimuli and adult stimuli) as within-subjects variables separately for reaction time as well as accuracy as the dependent variable. Verbal ability and gender of participant were included as covariates given the differences between the pubertal status groups in verbal ability and the unequal gender distribution (see **Table 1**).

Because pubertal groups differed with respect to age, it was also included as a covariate (similar to other studies, e.g., Picci and Scherf, 2016). Age was analyzed as a continuous variable (based on correlations with reaction times and error rate) as well as a categorical variable (“age group”). The correlation of age as a continuous measure and PDS was *r* = 0.63, *p* < 0.001; thus, below the commonly considered threshold of multicollinearity (*r* ≥ 0.7; Berry and Feldman, 1985; Dormann et al., 2013). Age group as a categorical variable was used in order to follow the approach of most previous studies exploring basic facial emotion recognition across puberty and adolescence that also grouped their participants in several distinct age groups to investigate development between these larger groups (Chronaki et al., 2015 using groups aged 4, 7, and 10 years; Herba et al., 2006: 6, 9, and 12 years; McGivern et al., 2002: 10, 11, 12, 13, 14, 15, and 16 years; Montirosso et al., 2010: 4–6, 7–9, 10–12, 13–15, and 16–18 years; Tonks et al., 2007: 9, 10, 11, 12, 13, and 14 years). For age group as a categorical variable we divided participants into five age groups (8–10, 11, 12, 13, and 16 years, see Supplementary Table S1). Importantly, the pattern of results remained comparable when we performed the same analyses without controlling for age group or covarying for age as a continuous measure (see Supplementary Table S6).

### Reaction Times

**FIGURE 2:**
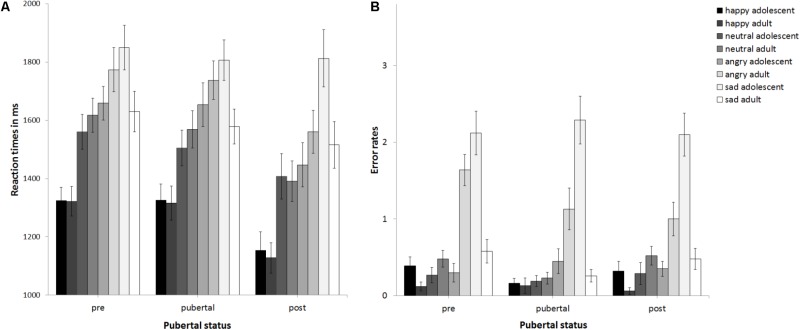
Mean reaction times **(A)** and error rates **(B)** as a function of pubertal status. Error bars indicate standard error of the mean (SEM).

**FIGURE 3:**
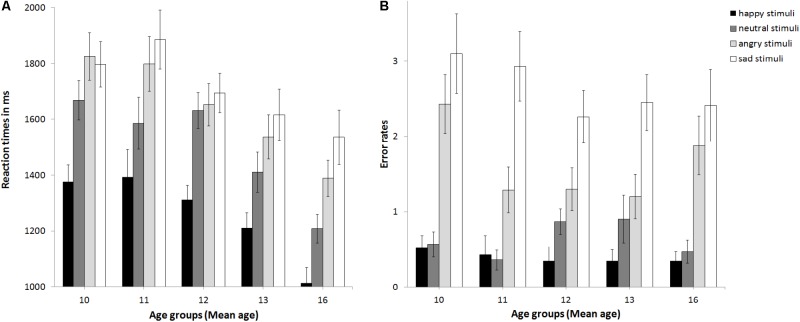
Mean reaction times **(A)** and error rates **(B)** as a function of age group. Error bars indicate SEM.

The ANCOVA with reaction times as the dependent variable (see **Figure 2A**) revealed no main effect of pubertal status, *F*(2,89) = 1.5, *p* = 0.23, partial η^2^ = 0.033, no main effect of stimulus age, *F*(1,89) = 0.19, *p* = 0.668, partial η^2^ = 0.002, but a main effect of emotion at trend, *F*(3,267) = 2.32, *p* = 0.076, partial η^2^ = 0.025. This was driven by sad being processed slowest, followed by angry, neutral, and happy, *p*s ≤ 0.031. The interaction of emotion × gender was significant, *F*(3,267) = 3.64, *p* = 0.013, partial η^2^ = 0.039. Girls (1240 ms) and boys (1291 ms) responded fastest to happy followed by neutral (boys: 1473 ms; girls: 1541 ms, *p* < 0.001). While for girls reaction times for angry (1602 ms) and sad stimuli (1670 ms) differed at trend, *p* = 0.073, there was no such difference for boys (angry: 1687 ms, sad: 1737 ms, *p* = 0.264). There were no other significant interactions, *p*s > 0.15. Thus, neither pubertal status nor stimulus age affected reaction times. Age group was a significant covariate, *F*(1,80) = 3.2, *p* = 0.001, partial η^2^ = 0.119. *Post hoc* *t*-tests (corrected for multiple testing using Bonferroni) regarding the effect of age group (see Supplementary Table S4 and **Figure 3A**) revealed that adolescents became faster from age group 11 and age group 12 to age group 16 years, *p* ≤ 0.02. No changes in reaction times were found between age group 8–10 and 11, or 12, or between age group 11 and 13, or 13 and 16, *p*s ≥0 .094. Verbal ability was also a significant covariate, *F*(1,89) = 6.15, *p* = 0.015, partial η^2^ = 0.065, with increasing verbal ability, reaction times became slower, *r*(93) = 0.42, *p* < 0.001. The covariate gender only trended toward significance *F*(1,89) = 3.2, *p* = 0.077, partial η^2^ = 0.035, with girls (1488 ms) performing slightly faster than boys (1543 ms; *p* = 0.397).

**FIGURE 4:**
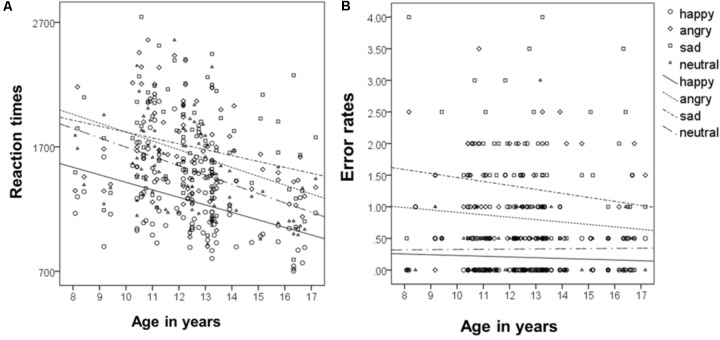
Correlation of mean reaction times **(A)** and error rates **(B)** with age as a continuous measure.

To analyze the relationship of type of emotion and age (following the approach of McGivern et al., 2002), correlations of age as a continuous measure and type of emotion for reaction times were calculated. This revealed that reaction times decreased for all emotions with increasing age, for happy *r*(93) = -0.4, for sad *r*(93) = -0.24, for angry, *r*(93) = -0.37, and for neutral, *r*(93) = -0.43 (all *p*s < 0.001, see **Figure 4A**). Overall reaction times independent of type of emotion, decreased across age, *r*(93) = -0.41 *p* < 0.001.

### Error Rates

The ANCOVA with error rates as the dependent variable (see **Figure 2B**) revealed that the main effect of pubertal status was not significant, *F*(2,89) = 0.96, *p* = 0.388, partial η^2^ = 0.021, as was the main effect of stimulus age, *F*(1,89) = 0.04, *p* = 0.838, partial η^2^ = 0. There was a trend for a main effect of emotion, *F*(2.31,205.73) = 2.88, *p* = 0.051, partial η^2^ = 0.031. *Post hoc* *t*-tests (corrected for multiple testing using Bonferroni) revealed that error rates differed between all emotions (*p* < 0.004) except for happy versus neutral (*p* = 0.2) and increased from 0.4 errors for happy over 0.66 errors for neutral and 1.63 for angry to 2.61 for sad. There was a trend for an interaction of emotion × stimulus age × age group, *F*(2.12,188.88) = 2.44, *p* = 0.087, partial η^2^ = 0.027. There were no other significant interactions, *ps* > 0.148. Thus, neither pubertal status nor stimulus age affected performance. The covariates gender, *F*(1,89) = 0.89, *p* = 0.348, partial η^2^ = 0.01, and verbal ability, *F*(1,89) = 0.2, *p* = 0.658 partial η^2^ = 0.002, were non-significant. There was a trend for the covariate age group, *F*(1,89) = 3.01, *p* = 0.086 partial η^2^ = 0.033. See also **Figure 3B** for descriptive effects regarding age group. Descriptively for angry faces error rates were higher for adult than adolescent stimuli, while for sad faces it was vice versa: error rates were higher for adolescent than adult stimuli (**Figure 2B**). Therefore, paired *t*-tests were calculated that revealed significant differences for angry adult (1.26 errors) vs. angry adolescent (0.37 errors) stimuli, *t*(94) = 5.91, *p* < 0.001, as well as for sad adult (0.44 errors) vs. sad adolescent (2.17 errors) stimuli, *t*(94) = -10.22, *p* < 0.001.

Correlations of age as a continuous measure and type of emotion for error rates revealed that error rates did not change significantly across age, neither for happy, *r*(93) = -0.06, sad, *r*(93) = -0.13, angry, *r*(93) = -0.1, nor for neutral, *r*(93) = 0.01 (all *ps* > 0.2, see **Figure 4B**). Overall error rates did also not change significantly across age, *r*(93) = -0.16 *p* = 0.121.

## Discussion

### Overview

This study aimed at investigating the “pubertal dip” phenomenon and tested whether there is an own-age bias, i.e., an enhanced processing of adolescent compared to adult emotional faces. We additionally explored whether specific emotions are recognized faster and more correctly than others in adolescents.

### No Evidence for a “Pubertal Dip”

In contrast to our hypothesis, we did not find any evidence for a pubertal dip in basic facial emotion recognition. Thus, this ability seems to be rather independent of pubertal status. Reaction times, instead, depended on age; adolescents get faster in judging basic facial emotions with increasing age (concordant to previous studies until age 13, De Sonneville et al., 2002; Rosenberg-Kima and Sadeh, 2010). In contrast, error rates did not depend on age. These findings are in contrast to the results of an increasing accuracy for some (negative) emotions (Thomas et al., 2007; Montirosso et al., 2010), and could be due to the study design of employing easier stimuli than earlier studies (e.g., stimuli with varying intensities or morphs). Importantly, participants’ performance was close to ceiling. This may indicate that the task was too easy, not sensitive enough and may have prevented the emergence of significant effects. Thus, based on performance levels alone, we cannot exclude the presence of a pubertal dip. However, reaction times (which are a more sensitive measure, Sheppard et al., 2015) also did not show a pubertal dip, but rather an increase in speed with increasing age which clearly argues against a pubertal dip. Moreover, variability in reaction times was similar as in the target study that found a pubertal dip in reaction times only (McGivern et al., 2002).

This is the first study that shows the lack of a pubertal dip, both in reaction times and error rates, while at the same time controlling for age group of adolescents and for a potential own-age bias of emotional stimuli. Our findings are in contrast to those of McGivern et al. (2002) who reported a pubertal dip in reaction times. Importantly, McGivern et al. (2002) defined the start of puberty by chronological age, while the current study used a self-report questionnaire. This study sampled pubertal groups *a priori* which presents a conceptual-methodical advantage compared to previous studies that classified pubertal status only *a posteriori* and then compared pubertal groups that differed in their sample size (McGivern et al., 2002; Tonks et al., 2007; Lawrence et al., 2008; Lawrence et al., 2015).

Conceptually, current findings add to two lines of evidence. First, face processing has been shown to underlie facial emotion processing both on a behavioral (e.g., explaining large variance in individual differences in emotion processing, Wilhelm, 2005; Hildebrandt et al., 2015) and a neural level (i.e., depending on similar routes of processing, Calder et al., 2001). Thus, our findings of no pubertal dip in basic facial emotion recognition dovetail nicely with the underlying ability of face recognition which also does not show a pubertal dip (Germine et al., 2011; Meinhardt-Injac et al., 2014). Second, there is concurrent evidence that the neural systems of emotion processing develop steadily (Crone and Elzinga, 2015) and thus support our finding of no pubertal disruption. Taken together, while only two previous studies have indicated the pubertal dip phenomenon in basic facial emotion recognition (McGivern et al., 2002; Tonks et al., 2007), current findings (together with conceptual evidence) provide contradictory evidence and indicate that the pubertal dip might not be a “genuine and reliable phenomenon” (Chung and Thomson, 1995, p. 62) for basic facial emotion recognition.

### No Evidence for an Own-Age Bias

Contrary to our hypothesis, basic facial emotion recognition was not superior for stimuli of the own-age group, i.e., adolescents; neither in terms of reaction times nor accuracy. Present findings are in line with recent studies in 7 and 10 year-old children (Griffiths et al., 2015) and in younger and older adults that also did not find any evidence for an own-age bias in basic facial emotion recognition (for a review see Fölster et al., 2014).

Overall, the own-age bias might be a more robust phenomenon for face recognition and not basic facial emotion recognition as found in Picci and Scherf (2016; for a review see also Rhodes and Anastasi, 2012). Alternatively, for adolescents, the own-age bias might rather be an own-pubertal-status bias as found in face recognition (Picci and Scherf, 2016). The current study (similar as others e.g., Griffiths et al., 2015) did not match stimuli in pubertal stage as Picci and Scherf (2016) did and therefore did not test this hypothesis. Future studies are needed to investigate and try to disentangle whether an own-age or an own-pubertal-status bias exists in basic facial emotion recognition or face recognition.

Similarly, to the interpretation of Griffiths et al. (2015) for children, adolescents might also have extensive experience with adults (parents, teachers, and older peers/young adults) and thus might not show lower performance in judging adult than peer faces. It could be interesting to try to quantify adolescents’ experience with different age groups and to investigate more fine-grained age ranges to further test this interpretation.

For angry faces error rates were higher for adult than adolescent stimuli, while for sad faces it was vice versa: error rates were higher for adolescent than adult stimuli. This might indicate that the stimuli from the adult and adolescent sets were not equally good representations of the emotions being conveyed.

However, the validation of the adolescent stimuli (Coffman et al., 2015) in an adolescent (*n* = 41, mean age = 14.54, *SD* = 1.7) and two adult sample s (*n* = 54 parents, modal age range 45–47; *n* = 34 health professionals: modal age range 50 and above) and the validation of the adult stimuli in an adult sample (*n* = 154, mean age = 49.83, age range 20–81 years; Ebner et al., 2010) do not correspond with this interpretation that stimuli in both sets were not equally good representations leading to the different error rates for adolescent vs. adult stimuli because accuracy differed slightly in the other direction: sad adolescent stimuli were rated better (84% adolescents/87% total accuracy) than adult stimuli (79%) and angry adult (91%) vs. adolescent stimuli (96% adolescents, 98% total, see Supplementary Tables S2, S3).

Another interpretation relates to findings that the recognition of anger depends on local, whereas the recognition of sadness depends on global facial features (Chiller-Glaus et al., 2011). This might interact with the clarity of (local and global) expression, which has been shown to differ between children and adults (Houstis and Kiliaridis, 2009). Overall, future studies are needed that address the question of an own-age bias across the life span and assess stimuli and participants from various age groups (children, adolescents, young, middle-aged, and old adults) while making sure that all stimuli convey the different emotions equally well.

### Effects of Type of Emotion

Consistent with our expectations, a differential performance in error rates and reaction times depending on type of emotion emerged. Performance was best for happy followed by neutral and angry, and worst for sad. As expected, we found that happy was recognized fastest and most correctly compared to other emotions across adolescence. This is in line with previous findings in children (Felleman et al., 1983; Kolb et al., 1992; Boyatzis and Chazan, 1993; Durand et al., 2007). The under researched expression of neutrality seemed to be more difficult to recognize and continues to develop across adolescence (Rodger et al., 2015). Finally, sadness seems to be more difficult to recognize than anger in adolescents which is consistent with other studies (Montirosso et al., 2010; Chronaki et al., 2015). However, the lack of significant differences between happy and neutral may also be due to the observed ceiling effects in these variables which makes it difficult to interpret the findings. Emotions of happiness and sadness are often among the first to be correctly recognized in early childhood, while the correct recognition of fear and disgust only develops during adolescence. Future studies should include a wider array of emotions (e.g., fear, sadness, anger, disgust, happiness, and surprise) and/or use morphed faces to make the task more difficult, to reduce ceiling levels of accuracy and observe a broader range of performance.

### Limitations

Although the pubertal groups were carefully matched in terms of non-verbal ability, socioeconomic status, and parental cultural capital, post-pubertal participants had a lower verbal ability than the other groups. It is unlikely that this caused the lacking pubertal dip phenomenon, since the dip should have resulted in a decrease of basic facial emotion recognition abilities from pre- to mid-puberty, which was not found. Second, the post-pubertal participants with lower verbal ability did not perform worse on basic facial emotion recognition than the other two groups, pubertal vs. post-pubertal group: *t*(60) = -4.16, *p* = 0.252; pre- vs. post-pubertal group: *t*(62) = 1.195, *p* = 0.237. On the contrary, reaction times decreased with age. Another limitation might be the unequal gender distribution, i.e., girls were overrepresented in the post-pubertal group. However, controlling for this variable did not change results. Although the sample size was appropriate for the research questions and similarly large as in other target studies (e.g., Chronaki et al., 2015; Griffiths et al., 2015), future studies are warranted that investigate the three main research questions in larger sample sizes.

Further limitations are related to the stimulus material. Given the lack of one overall stimulus set we took stimuli from different sets for adolescent and adult faces (similar as other studies had to, e.g., Griffiths et al., 2015; Picci and Scherf, 2016). Although we pilot tested our stimuli, we did not test systematically, e.g., whether both stimulus sets are conveying the emotions equally well in a sample that age-matched our participants (although both sets were well validated, Egger et al., 2011; Coffman et al., 2015). For future studies one large stimulus set is warranted that includes stimuli from different age groups and is tested on differently aged participants to ensure that emotions are equally well represented throughout the different stimulus age groups.

Although the adolescent faces with a mean age of 13.5 and a range of 10–16 fell within the age range of participants, it would be important for future studies to employ stimulus faces that more closely match the age of the participants like Griffiths et al. (2015) did (employing 5–8 and 9–12 years old stimuli and participants). However, also Griffiths et al. (2015) did not find evidence of an own-age bias.

Another approach to further test the own-pubertal-stage-bias would be to use faces that match participants’ pubertal status (and participants’ age, see Picci and Scherf, 2016). Taken together, stimuli could match the age AND/OR pubertal status of participants in future studies (e.g., 14-year old participant has to rate 14-year old stimuli; mid-pubertal participant has to rate mid-pubertal stimuli; 14-year old mid-pubertal participant has to rate 14-year old stimuli that are mid-pubertal). Although methodically very challenging, such a design could more specifically test the influence of age and pubertal status on emotion recognition and potentially try to disentangle these two different influences.

Given the limitations of a self-report questionnaire, a direct measurement of pubertal status such as hormone levels and physician ratings may help to cross-validate current findings in future studies. However, the PDS represents a widely used measure, e.g., in the target studies on emotional abilities (Keulers et al., 2010; Vetter et al., 2013; Lawrence et al., 2015), and is in moderate agreement with clinician-ratings and related to basal hormones (Shirtcliff et al., 2009; Saxbe et al., 2014). It also has to be noted that for physical ratings more effort from participants is required and given it is a sensitive topic participants have been shown to refuse the exam while assenting to fill in the PDS (Shirtcliff et al., 2009).

## Conclusion

The current study points to no pubertal dip in basic facial emotion recognition in error rates and reaction times. Instead, with increasing age adolescents seem to become faster in basic facial emotion recognition. Furthermore, there was no own-age bias for basic facial emotion recognition in adolescents. Overall, these findings imply no disruption of basic facial emotion recognition at the beginning of puberty but a continuous linear development for speed and stability for accuracy across adolescence. Longitudinal studies are further warranted to more precisely investigate the role of pubertal change and emotional skills. A better characterization of the developmental trajectory of emotional skills across adolescence and puberty might help to understand the emotional challenges during adolescence, including adequate reactions toward peers, teachers, and parents in emotion-laden situations. The description of typical development will also help to better understand affective disorders that often emerge in adolescence.

## Ethics Statement

This study was carried out in accordance with the recommendations of the ‘Ethikkommission an der TU Dresden’ with written informed consent from all subjects. All subjects gave written informed consent in accordance with the Declaration of Helsinki. The protocol was approved by the ‘Ethikkommission an der TU Dresden.’

## Author Contributions

MD and JT carried out the data acquisition. NV, MD, and JT performed the data analysis. NV drafted the manuscript. All authors contributed to the experimental design of the study, were involved in the interpretation of data, revised the manuscript critically, approved the submitted version to be published, and hold themselves accountable for all aspects of the work in ensuring that questions related to the accuracy or integrity of any part of the work were appropriately investigated and resolved.

## Conflict of Interest Statement

The authors declare that the research was conducted in the absence of any commercial or financial relationships that could be construed as a potential conflict of interest.

## Footnotes

1 https://pstnet.com/products/e-prime/
