# Supplementary material for: Adolescent Basic Facial Emotion Recognition Is Not Influenced by Puberty or Own-Age Bias
Source: Front Psychol. 2018 Jun 21;9:956. doi: 10.3389/fpsyg.2018.00956 (PMC6022279; doi:10.3389/fpsyg.2018.00956)
Supplement: Supplementary file 1 [file Table_1.docx]

# **Supporting Information**

S1 Table. Descriptive data for age groups with means and (standard deviations).

| Age range of group in years | 8-10 | 11 | 12 | 13 | 14-17 |
| --- | --- | --- | --- | --- | --- |
| N | 21 | 14 | 29 | 20 | 19 |
| Age | 10.06 (0.92) | 11.32 (0.3) | 12.42 (0.26) | 13.29 (0.21) | 15.74 (1.03) |
| Number of Females | 16 | 8 | 10 | 10 | 8 |
| Pubertal status | 1.38 (0.59) | 1.5 (0.65) | 1.78 (0.74) | 2.4 (0.68) | 2.93 (0.26) |
| Socioeconomic status ^a^ | 13.29 (4.33) | 13.39 (4.72) | 14.00 (3.82) | 14.63 (4.04) | 14.9 (4.07) |
| Numbers ^b^ | 10.71 (2.31) | 10.86 (2.56) | 11.61 (2.81) | 10.55 (2.7) | 10.88 (2.52) |
| Verbal abilities ^b^ | 12.81 (2.11) | 13.57 (2.03) | 12.57 (1.81) | 11.4 (2.33) | 10.07 (1.28) |

*Notes.* ^a^ as suggested by Winkler and Stolzenberg (2009) ranging from 3 to 21 with higher values indicating higher socioeconomic status; 1 = lower class, 2 = middle class, 3 = upper class (De Graaf et al., 2000);

^b^ assessed by the Vocabulary subtest score of the Wechsler Intelligence Scale For Children (WISC-IV, German adaptation, Petermann & Petermann, 2007).
