# Supplementary material for: Adolescent Basic Facial Emotion Recognition Is Not Influenced by Puberty or Own-Age Bias
Source: Front Psychol. 2018 Jun 21;9:956. doi: 10.3389/fpsyg.2018.00956 (PMC6022279; doi:10.3389/fpsyg.2018.00956)
Supplement: Supplementary file 2 [file Table_2.docx]

# **Supporting Information**

**S2 Table. Features of the adolescent pictures of the NIMH-ChEFS sorted by stimulus type and agreement (taken from supplemental information of Egger et al., 2011 and Coffman et al., 2015).**

| Filename | Stimulus type | Actor’s Age | Agreement  (from n=0 to 20 raters) | Intensity ^a^  (-100 – 100) | Representativeness  (-100 – 100) | Goodness ^a^  (-100 – 100) | Accuracy Average Teens (0-1) | Accuracy Average Total (0-1) |
| --- | --- | --- | --- | --- | --- | --- | --- | --- |
| F1AS_3411.jpg | angry_direct_female | 14 | 20 | 78 | 76 | 61 | 1.00 | 1.00 |
| F2AS_3506.jpg | angry_direct_female | 14 | 20 | 84 | 80 | 69 | 1.00 | 1.00 |
| F9AS_4247.jpg | angry_direct_female | 13 | 20 | 70 | 81 | 58 | 1.00 | 1.00 |
| F31AS_7888.jpg | angry_direct_female | 15 | 20 | 76 | 82 | 63 | 1.00 | 0.99 |
| F35AS_8470.jpg | angry_direct_female | 14 | 19 | 65 | 64 | 49 | 1.00 | 1.00 |
| M3AS_4811.jpg | angry_direct_male | 15 | 18 | 61 | 53 | 42 | 0.82 | 0.94 |
| M7AS_5550.jpg | angry_direct_male | 16 | 19 | 65 | 60 | 47 | 0.96 | 0.97 |
| M10AS_6840.jpg | angry_direct_male | 12 | 19 | 65 | 63 | 46 | 0.93 | 0.95 |
| M12AS_7102.jpg | angry_direct_male | 11 | 20 | 65 | 68 | 47 | 0.92 | 0.98 |
| M20AS_9297.jpg | angry_direct_male | 13 | 20 | 57 | 65 | 39 | 0.92 | 0.95 |
|  | **Angry overall mean** | **13.7** | **19.50** | **68.60** | **69.20** | **52.10** | **0.96** | **0.98** |
|  | **Angry overall mean %** | **-** | **97.5** | **84.3** | **84.6** | **76.1** | **95.6** | **97.8** |
| F4HS_3664.jpg | happy_direct_female | 15 | 20 | 77 | 84 | 66 | 1.00 | 1.00 |
| F12HS_5000.jpg | happy_direct_female | 12 | 20 | 77 | 89 | 69 | 1.00 | 1.00 |
| F20HS_6413.jpg | happy_direct_female | 14 | 20 | 77 | 87 | 68 | 1.00 | 1.00 |
| F34HS_8209.jpg | happy_direct_female | 14 | 20 | 57 | 64 | 40 | 0.96 | 0.99 |
| F37HS_8954.jpg | happy_direct_female | 12 | 20 | 66 | 71 | 53 | 1.00 | 1.00 |
| M4HS_4862.jpg | happy_direct_male | 14 | 20 | 80 | 81 | 66 | 1.00 | 1.00 |
| M6HS_5354.jpg | happy_direct_male | 14 | 20 | 80 | 89 | 73 | 1.00 | 1.00 |
| M9HS_6054.jpg | happy_direct_male | 13 | 20 | 69 | 76 | 54 | 0.96 | 0.99 |
| M12HS_7022.jpg | happy_direct_male | 11 | 20 | 64 | 80 | 52 | 1.00 | 1.00 |
| M19HS_9059.jpg | happy_direct_male | 12 | 20 | 61 | 79 | 49 | 1.00 | 1.00 |
|  | **Happy overall Mean** | **13.1** | **20.00** | **80.00** | **81.00** | **66.00** | **0.99** | **1.00** |
|  | **Happy overall Mean %** | **-** | **100.0** | **90.0** | **90.5** | **83.0** | **99.3** | **99.8** |
| F11NS_4515.jpg | neutral_direct_female | 14 | 19 | 48 | 73 | 41 | 0.89 | 0.94 |
| F13NS_5249.jpg | neutral_direct_female | 14 | 19 | 56 | 65 | 41 | 1.00 | 1.00 |
| F25NS_5531.jpg | neutral_direct_female | 13 | 18 | 49 | 64 | 40 | 1.00 | 0.97 |
| F36NS_8495.jpg | neutral_direct_female | 14 | 20 | 60 | 77 | 47 | 1.00 | 1.00 |
| F33NS_8095.jpg | neutral_direct_female | 14 | 20 | 54 | 69 | 41 | 1.00 | 0.93 |
| M11NS_6873.jpg | neutral_direct_male | 11 | 20 | 59 | 75 | 47 | 1.00 | 0.86 |
| M16NS_8625.jpg | neutral_direct_male | 13 | 20 | 53 | 67 | 36 | 0.93 | 0.95 |
| M17NS_8725.jpg | neutral_direct_male | 10 | 20 | 57 | 72 | 43 | 0.92 | 0.93 |
| M18NS_8844.jpg | neutral_direct_male | 16 | 20 | 53 | 69 | 38 | 0.92 | 0.88 |
| M8NS_5807.jpg | neutral_direct_male | 16 | 18 | 39 | 55 | 28 | 0.92 | 0.95 |
|  | **Neutral overall mean** | **13.4** | **19.40** | **52.80** | **68.60** | **40.20** | **0.96** | **0.94** |
|  | **Neutral overall mean %** | **-** | **97.0** | **76.4** | **84.3** | **70.1** | **95.9** | **94.1** |
| F11SS_4544.jpg | sad_direct_female | 14 | 20 | 58 | 57 | 35 | 1.00 | 0.95 |
| F13SS_5276.jpg | sad_direct_female | 14 | 20 | 69 | 76 | 57 | 0.92 | 0.98 |
| F15SS_5712.jpg | sad_direct_female | 15 | 18 | 43 | 54 | 31 | 0.79 | 0.92 |
| F19SS_6316.jpg | sad_direct_female | 13 | 19 | 62 | 67 | 48 | 1.00 | 0.98 |
| F36SS_8532.jpg | sad_direct_female | 14 | 20 | 49 | 61 | 33 | 0.85 | 0.90 |
| M4SS_4889.jpg | sad_direct_male | 14 | 18 | 38 | 43 | 23 | 0.69 | 0.76 |
| M7SS_5508.jpg | sad_direct_male | 16 | 20 | 53 | 67 | 37 | 0.69 | 0.76 |
| M12SS_7038.jpg | sad_direct_male | 11 | 15 | 29 | 30 | 19 | 0.69 | 0.67 |
| M14SS_7930.jpg | sad_direct_male | 15 | 19 | 55 | 52 | 39 | n. s.^b^ | n. s.^b^ |
| M19SS_9083.jpg | sad_direct_male | 12 | 20 | 54 | 61 | 36 | 0.93 | 0.95 |
|  | **Sad overall mean** | **13.8** | **18.90** | **51.00** | **56.80** | **35.80** | **0.84** | **0.87** |
|  | **Sad overall mean %** | **-** | **94.5** | **75.5** | **78.4** | **67.9** | **84.0** | **87.5** |
| *Notes.* ^a^ According to Egger et al. (2011) not interpretable for neutral stimuli; ^b^ not specified. | | | | | | | | |
