# Supplementary material for: Adolescent Basic Facial Emotion Recognition Is Not Influenced by Puberty or Own-Age Bias
Source: Front Psychol. 2018 Jun 21;9:956. doi: 10.3389/fpsyg.2018.00956 (PMC6022279; doi:10.3389/fpsyg.2018.00956)
Supplement: Supplementary file 3 [file Table_3.DOCX]

# **Supporting Information**

S3 Table. Features of the adult pictures of the FACES database including accuracy rates in a validation sample (taken from supplemental information of Ebner et al., 2010).

| Filename | Stimulus type | Mean % Accuracy |
| --- | --- | --- |
| 006_m_f_a_b.jpg | angry_female | 95 |
| 061_m_f_a_b.jpg | angry_female | 87 |
| 113_m_f_a_b.jpg | angry_female | 94 |
| 168_m_f_a_b.jpg | angry_female | 91 |
| 171_y_f_a_a.jpg | angry_female | 97 |
| 032_m_m_a_b.jpg | angry_male | 89 |
| 058_m_m_a_a.jpg | angry_male | 70 |
| 144_y_m_a_b.jpg | angry_male | 94 |
| 155_m_m_a_b.jpg | angry_male | 98 |
| 175_y_m_a_b.jpg | angry_male | 97 |
|  | **Angry overall** | **91.2** |
| 029_m_f_h_a.jpg | happy_female | 97 |
| 073_m_f_h_a.jpg | happy_female | 100 |
| 084_m_f_h_b.jpg | happy_female | 98 |
| 117_m_f_h_a.jpg | happy_female | 100 |
| 156_m_f_h_b.jpg | happy_female | 100 |
| 014_m_m_h_b.jpg | happy_male | 97 |
| 045_m_m_h_a.jpg | happy_male | 97 |
| 068_m_m_h_b.jpg | happy_male | 94 |
| 136_m_m_h_a.jpg | happy_male | 100 |
| 179_m_m_h_b.jpg | happy_male | 95 |
|  | **Happy overall** | **97.8** |
| 019_m_f_n_a.jpg | neutral_female | 78 |
| 080_m_f_n_b.jpg | neutral_female | 85 |
| 113_m_f_n_b.jpg | neutral_female | 96 |
| 138_m_f_n_a.jpg | neutral_female | 94 |
| 171_y_f_n_b.jpg | neutral_female | 91 |
| 026_m_m_n_b.jpg | neutral_male | 97 |
| 038_m_m_n_a.jpg | neutral_male | 94 |
| 108_m_m_n_a.jpg | neutral_male | 92 |
| 116_m_m_n_b.jpg | neutral_male | 91 |
| 165_m_m_n_b.jpg | neutral_male | 94 |
|  | **Neutral overall** | **91.2** |
| 011_m_f_s_b.jpg | sad_female | 46 |
| 043_m_f_s_b.jpg | sad_female | 89 |
| 084_m_f_s_a.jpg | sad_female | 78 |
| 093_m_f_s_a.jpg | sad_female | 77 |
| 139_m_f_s_a.jpg | sad_female | 79 |
| 007_m_m_s_a.jpg | sad_male | 71 |
| 045_m_m_s_a.jpg | sad_male | 93 |
| 068_m_m_s_a.jpg | sad_male | 84 |
| 160_y_m_s_b.jpg | sad_male | 92 |
| 169_m_m_s_a.jpg | sad_male | 84 |
|  | **Sad overall** | **79.3** |
