# Supplementary material for: Adolescent Basic Facial Emotion Recognition Is Not Influenced by Puberty or Own-Age Bias
Source: Front Psychol. 2018 Jun 21;9:956. doi: 10.3389/fpsyg.2018.00956 (PMC6022279; doi:10.3389/fpsyg.2018.00956)
Supplement: Supplementary file 4 [file Table_4.docx]

# **Supporting Information**

**S4 Table. Descriptive results of mean reaction times and mean error rates for age groups ± standard deviations.**

|  | | Age group 1 (10 y) | Age group 2 (11 y) | Age group 3 (12 y) | Age group 4 (13 y) | Age group 5 (16 y) |
| --- | --- | --- | --- | --- | --- | --- |
| Reaction times | angry adult | 1889 ± 428 | 1881 ± 460 | 1658 ± 370 | 1582 ± 365 | 1472 ± 326 |
|  | happy adult | 1364 ± 294 | 1391 ± 424 | 1324 ± 258 | 1204 ± 242 | 986 ± 199 |
|  | neutral adult | 1676 ± 330 | 1613 ± 351 | 1663 ± 377 | 1426 ± 338 | 1213 ± 230 |
|  | sad adult | 1648 ± 394 | 1763 ± 429 | 1626 ± 337 | 1502 ± 393 | 1354 ± 360 |
|  | angry adolescent | 1761 ± 412 | 1714 ± 343 | 1646 ± 402 | 1492 ± 381 | 1307 ± 301 |
|  | happy adolescent | 1389 ± 308 | 1395 ± 343 | 1299 ± 284 | 1218 ± 277 | 1039 ± 275 |
|  | neutral adolescent | 1660 ± 392 | 1559 ± 370 | 1599 ± 321 | 1396 ± 357 | 1202 ± 289 |
|  | sad adolescent | 1945 ± 429 | 2006 ± 443 | 1763 ± 379 | 1731 ± 472 | 1717 ± 550 |
|  | all stimuli | 1666 ± 289 | 1665 ± 337 | 1572 ± 267 | 1444 ± 300 | 1286 ± 217 |
| Error rates | angry adult | 2.00 ± 1.61 | 1.14 ± 1.10 | 0.91 ± 1.13 | 1.00 ± 1.08 | 1.24 ± 1.39 |
|  | happy adult | 0.10 ± 0.30 | 0.00 ± 0.00 | 0.17 ± 0.65 | 0.15 ± 0.37 | 0.06 ± 0.24 |
|  | neutral adult | 0.38 ± 0.67 | 0.36 ± 0.50 | 0.48 ± 0.59 | 0.45 ± 0.69 | 0.35 ± 0.49 |
|  | sad adult | 0.62 ± 0.80 | 0.14 ± 0.36 | 0.39 ± 0.72 | 0.50 ± 0.89 | 0.47 ± 0.62 |
|  | angry adolescent | 0.43 ± 0.75 | 0.14 ± 0.36 | 0.39 ± 0.72 | 0.20 ± 0.52 | 0.65 ± 1.00 |
|  | happy adolescent | 0.43 ± 0.68 | 0.43 ± 0.94 | 0.17 ± 0.39 | 0.20 ± 0.52 | 0.29 ± 0.47 |
|  | neutral adolescent | 0.19 ± 0.51 | 0.00 ± 0.00 | 0.39 ± 0.58 | 0.45 ± 0.95 | 0.12 ± 0.33 |
|  | sad adolescent | 2.48 ± 2.06 | 2.79 ± 1.72 | 1.87 ± 1.52 | 1.95 ± 1.15 | 1.94 ± 1.56 |
|  | all stimuli | 6.62 ± 3.03 | 5.00 ± 2.83 | 4.78 ± 2.66 | 4.90 ± 2.47 | 5.12 ± 2.47 |
