# Supplementary material for: Adolescent Basic Facial Emotion Recognition Is Not Influenced by Puberty or Own-Age Bias
Source: Front Psychol. 2018 Jun 21;9:956. doi: 10.3389/fpsyg.2018.00956 (PMC6022279; doi:10.3389/fpsyg.2018.00956)
Supplement: Supplementary file 5 [file Table_5.DOCX]

# **Supporting Information**

**S5 Table. Descriptive results of mean reaction times and mean error rates for pubertal groups ± standard deviations.**

|  |  | Pre-pubertal | Pubertal | Post-pubertal |
| --- | --- | --- | --- | --- |
| Reaction times | angry adult | 1774 ± 437 | 1738 ± 371 | 1561 ± 410 |
|  | happy adult | 1322 ± 290 | 1316 ± 327 | 1128 ± 290 |
|  | neutral adult | 1618 ± 333 | 1569 ± 359 | 1391 ± 387 |
|  | sad adult | 1630 ± 399 | 1579 ± 334 | 1516 ± 446 |
|  | angry adolescent | 1659 ± 332 | 1654 ± 421 | 1447 ± 423 |
|  | happy adolescent | 1325 ± 263 | 1326 ± 309 | 1154 ± 350 |
|  | neutral adolescent | 1561 ± 345 | 1505 ± 343 | 1408 ± 436 |
|  | sad adolescent | 1850 ± 440 | 1807 ± 386 | 1813 ± 548 |
|  | all stimuli | 1592 ± 273 | 1562 ± 290 | 1427 ± 348 |
| Error rates | angry adult | 1.64 ± 1.17 | 1.13 ± 1.52 | 1.00 ± 1.21 |
|  | happy adult | 0.12 ± 0.33 | 0.13 ± 0.56 | 0.06 ± 0.25 |
|  | neutral adult | 0.48 ± 0.62 | 0.23 ± 0.43 | 0.52 ± 0.68 |
|  | sad adult | 0.58 ± 0.87 | 0.26 ± 0.45 | 0.48 ± 0.77 |
|  | angry adolescent | 0.30 ± 0.68 | 0.45 ± 0.89 | 0.35 ± 0.55 |
|  | happy adolescent | 0.39 ± 0.66 | 0.16 ± 0.37 | 0.32 ± 0.70 |
|  | neutral adolescent | 0.27 ± 0.57 | 0.19 ± 0.40 | 0.29 ± 0.78 |
|  | sad adolescent | 2.12 ± 1.64 | 2.29 ± 1.74 | 2.10 ± 1.56 |
|  | all stimuli | 5.91 ± 2.69 | 4.84 ± 2.96 | 5.13 ± 2.53 |
