# Supplementary material for: Adolescent Basic Facial Emotion Recognition Is Not Influenced by Puberty or Own-Age Bias
Source: Front Psychol. 2018 Jun 21;9:956. doi: 10.3389/fpsyg.2018.00956 (PMC6022279; doi:10.3389/fpsyg.2018.00956)
Supplement: Supplementary file 6 [file Table_6.docx]

# **Supporting Information**

**S6. Alternative ANCOVAS for reaction times and error rates.**

**Reaction times**

1. **ANCOVA reaction times with the covariate age as a continuous variable**

The ANCOVA with reaction times as the dependent variable revealed no main effect of pubertal status, *F*(2,89) = .65, *p =* .524, partial *η2* = .014, no main effect of stimulus age, *F*(1,89) = .34, *p =* .562, partial *η2* = .004, and no main effect of emotion, *F*(3,267) = .87, *p =* .458, partial *η2* = .01. Emotion x gender was a significant interaction, *F*(3,267) = 2.92, *p =* .035, partial *η2* = .032, which was driven by girls (1240 ms) and boys (1291 ms) being fastest for happy followed by neutral (boys: 1473 ms; girls: 1541 ms, p < .001), but for girls this was followed by angry (1602 ms) and sad (1670 ms), differing at trend, p = .073 while for boys RTs for angry (1687 ms) and sad (1737 ms) did not differ from each other, p = .264. There were no other significant interactions, all *p*s > .093. Age, *F*(1,89) = 7.86, *p* = .006, partial *η2* = .081, and verbal ability, *F*(1,89) = 7.18, *p* = .009, partial *η2* = .075, were significant covariates but not gender *F*(1,89) = 1.69, *p* = .198, partial *η2* = .019.

1. **ANCOVA reaction times without the covariate age as a continuous or categorical variable**

The ANCOVA with reaction times as the dependent variable revealed no main effect of pubertal status, *F*(2,90) = .46, *p =* .632, partial *η2* = .01, no main effect of stimulus age, *F*(1,90) = .118, *p =* .732, partial *η2* = .001, and a trend for a main effect of emotion, *F*(3,270) = 2.6, *p =* .053, partial *η2* = .028. This was driven by sad being processed slowest, followed by angry, neutral, and happy, *p*s ≤ .031. Emotion x gender was a significant interaction, *F*(3,270) = 3.48, *p =* .016, partial *η2* = .037, which was driven by girls (1240 ms) and boys (1291 ms) being fastest for happy followed by neutral (boys: 1473 ms; girls: 1541 ms, p < .001), but for girls this was followed by angry (1602 ms) and sad (1670 ms), differing at trend, p = .073 while for boys RTs for angry (1687 ms) and sad (1737 ms) did not differ from each other, p = .264. For emotion x verbal ability there was a trend, *F*(3,270) = 2.42, *p =* .067, partial *η2* = .026. There were no other significant interactions, all *p*s > .0233. Verbal ability, *F*(1,90) = 14.86, *p* < .001, partial *η2* = .142, was a significant covariate but not gender *F*(1,90) = .002, *p* = .96, partial *η2* = 0.

**Error rates**

1. **ANCOVA error rates with the covariate age as a continuous variable**

The ANCOVA with error rates as the dependent variable revealed that the main effect of pubertal status was non-significant, *F*(2,89) = .9, *p =* .408, partial *η2* = .02 as was the main effect of stimulus age, *F*(1,89) = .023, *p =* .897, partial *η2* = 0. There was a trend of a main effect of emotion, *F*(2.29,206.68) = 2.29, *p =* .096, partial *η2* = .025. Post-hoc t-tests (corrected for multiple testing) revealed that error rates differed between all emotions (*p* < .004) except for happy versus neutral (*p* = .2) and increased from 0.4 errors for happy over 0.66 errors for neutral and 1.63 for angry to 2.61 for sad. There were no significant interactions, *p*s > .232. The covariates gender, *F*(1,89) = .5, *p* = .482, partial *η2* = .01, and verbal ability, *F*(1,89) = .1, *p* = .753 partial *η2* = .001, as well as age, *F*(1,89) = 2.01, *p* = .159 partial *η2* = .022, were non-significant.

1. **ANCOVA error rates without the covariate age as a continuous or categorical variable**

The ANCOVA with error rates as the dependent variable revealed that the main effect of pubertal status was non-significant, *F*(2,90) = 1.21, *p =* .304, partial *η2* = .026 as was the main effect of stimulus age, *F*(1,90) = .094, *p =* .76, partial *η2* = .001. There was no main effect of emotion, *F*(2.3,207.05) = 1.09, *p =* .354, partial *η2* = .012. There was a trend for the interaction of stimulus age x emotion x verbal ability, *F*(2.55,189.72) = 2.55, *p =* .077, partial *η2* = .028. There were no other significant interactions, *p*s > .157. The covariates gender, *F*(1,90) = .006, *p* = .937, partial *η2* = .0, and verbal ability, *F*(1,90) = .05, *p* = .823 partial *η2* = .001, were non-significant.
